# Implications of Skeletal Muscle Extracellular Matrix Remodeling in Metabolic Disorders: Diabetes Perspective

**DOI:** 10.3390/ijms21113845

**Published:** 2020-05-28

**Authors:** Khurshid Ahmad, Inho Choi, Yong-Ho Lee

**Affiliations:** 1 Department of Medical Biotechnology, Yeungnam University, Gyeongsan 38541, Korea; ahmadkhursheed2008@gmail.com (K.A.); inhochoi@ynu.ac.kr (I.C.); 2 Department of Biomedical Science, Daegu Catholic University, Gyeongsan 38430, Korea

**Keywords:** extracellular matrix, remodeling, insulin resistance, integrin, diabetes

## Abstract

The extracellular matrix (ECM) provides a scaffold for cells, controlling biological processes and providing structural as well as mechanical support to surrounding cells. Disruption of ECM homeostasis results in several pathological conditions. Skeletal muscle ECM is a complex network comprising collagens, proteoglycans, glycoproteins, and elastin. Recent therapeutic approaches targeting ECM remodeling have been extensively deliberated. Various ECM components are typically found to be augmented in the skeletal muscle of obese and/or diabetic humans. Skeletal muscle ECM remodeling is thought to be a feature of the pathogenic milieu allied with metabolic dysregulation, obesity, and eventual diabetes. This narrative review explores the current understanding of key components of skeletal muscle ECM and their specific roles in the regulation of metabolic diseases. Additionally, we discuss muscle-specific integrins and their role in the regulation of insulin sensitivity. A better understanding of the importance of skeletal muscle ECM remodeling, integrin signaling, and other factors that regulate insulin activity may help in the development of novel therapeutics for managing diabetes and other metabolic disorders.

## 1. Introduction

Extracellular matrix (ECM) is a multifaceted meshwork of proteins surrounding cells, essentially providing mechanical support for cells to remain intact, as well as facilitating communication between cells [1]. ECM proteins are major components of nearly every tissue type. ECM helps arrange cells to modulate several essential biological processes, such as proliferation, adhesion, migration, and differentiation. ECM components are broadly classified into different families, including collagens, laminins, and proteoglycans [2]. The ECM has been recognized as a fundamental intermediary in diverse cellular mechanisms, and ECM cross-linking and proteolytic cascades appear to be important in both disease and health. Maintaining the homeostasis of ECM is important for human health, since altered homeostasis results in several metabolic disorders and other pathological conditions. The ECM is gaining the interest of researchers as a subject of intensive research across all organ systems for the investigation of novel disease mechanisms, diagnostic markers, and drug targets related to the ECM and its components [3,4]. Therapeutic approaches for ECM-related pathological conditions, as well as ECM remodeling, have been broadly considered recently [5].

Skeletal muscle (SM), the largest tissue by mass in humans (nearly 40%), is composed largely of myofiber and provides movement and support to the skeleton, as well as controlling body temperature. In addition, SM assists in regulating several physiological processes, including metabolism and myokine secretion, and communicates with other tissues [6]. SM is an essential metabolic tissue responsible for the majority of insulin-mediated glucose uptake (nearly 85%) via glucose transporter type 4 (GLUT4) [7]. SM stem cells, also called muscle satellite cells (MSCs) are positioned between the sarcolemma and basal lamina of muscle fibers and have a fundamental role in muscle regeneration. MSCs are normally found in an inactive form and remain dormant until injury or exercise [8]. The basal lamina is composed of non-fibrillar collagen, proteoglycans, and noncollagenous glycoproteins, creating a network for the ECM [9].

SM ECM is extremely flexible and therefore its texture and functional efficiency contributes to the regulation of physiological processes and may be affected by several factors, including aging and pathological conditions such as type 2 diabetes mellitus (T2DM) [10]. Although many studies have focused on skeletal muscle for the therapeutic management of metabolic disorders, especially T2DM, there are limited studies on targeting the SM ECM and its remodeling [11]. This review aims to encourage further research into the remodeling of major SM ECM components responsible for metabolic disorders. We provide a brief introduction and current status of knowledge on the SM ECM and its major components that are directly or indirectly involved in disease conditions related to metabolism and T2DM.

## 2. Skeletal Muscle ECM: Composition, Structure and Function

SM ECM is composed of several proteoglycans and fibrous proteins, including collagens, fibronectin, laminins, and elastins [12]. Collagen is the key component of fibrous protein in SM ECM, accounting for up to 10% of SM by weight and forming a network of intramuscular connective tissue (IMCT) [12]. IMCT is organized into endomysium (inner), perimysium, and epimysium (outer) layers. Collagen I and III are abundant in the IMCT, while other types of collagens are also present [13].

Collagen I is the main component of epimysium and perimysium, while collagen III is a minor component. Both collagen I and III are the major components of the endomysium [14]. The endomysium shares a boundary with the myofiber sarcolemma at the basal lamina of the basement membrane (BM). The BM is composed of the reticular lamina and basal lamina, which contain collagen IV, nidogen, perlecan, and laminins [15]. Collagen VI, XV, and XVIII are also components of SM BM [16]. Laminins are the key components of the SM BM surrounding muscle fibers and stimulate proliferation, migration, differentiation, and survival of myoblasts [17]. Laminin binds with its transmembrane receptors dystroglycan and integrin α7β1 in the SM. There are several isoforms of laminins, and among them, laminin-211 is the most abundant isoform found in the adult SM BM [18,19].

We have explored ECM components in the context of their prominent roles in SM at different stages of development and during regeneration processes [20,21,22]. The role of fibromodulin and matrix gla protein during myoblast differentiation is to regulate the interaction of myostatin, a well-known negative regulator of myogenesis, with activin receptor type IIB (ACVRIIB). These findings suggest possibilities for innovative therapeutics that may prove beneficial in the treatment of several muscle-related diseases [22,23,24]. Recently, we explored the role of dermatopontin (DPT), a non-collagenous ECM component dynamically involved in myogenesis, finding that it increases cell adhesion, decreases proliferation, and enhances differentiation [20]. In another study, DPT increased expression of ECM-related genes (COL6A3, TNMD, MMP-9, and ELN) and inflammation-associated factors (TNF, IL-6, and IL-8) in human visceral adipocytes, indicating a role for DPT in the regulation of ECM remodeling [25].

### 2.1. Collagen

Collagens are the most abundant protein in multicellular organisms and are the major component of the ECM [26]. The collagen gene family consists of 46 genes encoding 28 different types of proteins comprising different combinations; each type of collagen protein consists of homo- and hetero-trimers made of three polypeptide chains [27]. Collagens are the most prominent ECM component in SM as they play a crucial role in the regulation of cell attachment and differentiation, providing elasticity and tensile strength to bones [1]. There are 28 diverse forms of collagen, of which 11 types (collagen I, III, IV, V, VI, XII, XIII, XIV, XV, XVIII, and XXII) have been identified in mature SM [1,10]. Collagen I, III, V, IX, and XI are the fibrillar collagens found in SM, with I and III types being the most abundant, accounting for almost 75% of total SM collagen [13]. Collagen IV, a triple-helical molecule, is the key structural component of the basal lamina. Both fibrogenic and myogenic cells are known to secrete collagen IV [28].

Collagen type I alpha 1 and 2 chains (COL1A1/2) are fibrillar collagens found in all the three (endo-, peri-, and epimysium) layers; COL1A1/2 forms parallel fibers and determines tensile strength and rigidity in SM. Collagen type III alpha 1 chain (COL3A1) is also a fibrillar collagen that appears in the endo- and perimysium as well as the myotendinous junction and forms a loose meshwork of fibers [29]. Collagen type V alpha 1–3 chains (COL5A1–3) controls fibrillogenesis [29]. Collagen type VI alpha 1–6 chains (COL6A1–6) are the main beaded filaments: the α6 chain is predominantly found in endo- and perimysium; the α3 chain in basal lamina; and the α5 chain in the myotendinous junction (MTJ) [30]. Collagen VI is known to interact with several ECM components and cell surface receptors, and has a significant role in maintaining the functional integrity of the SM. Mutations in the *COL6A1*, *COL6A2*, and *COL6A3* genes are associated with muscle disorders namely Ullrich congenital muscular dystrophy and Bethlem myopathy [31]. Collagen type XV alpha 1 chain (COL15A1), collagen type XVIII alpha 1 chain (COL18A1), and collagen type XIX alpha 1 chain (COL19A1) are components of the BM of SM [10,32]. Collagen type XXII alpha 1 chain (COL22A1) is found in the MTJ and assimilates ECM components, providing mechanical stability to the MTJ. The knockdown of the *COL22A1* gene results in muscular dystrophy in zebrafish by disruption of the MTJ [33,34].

Generally, collagen is produced by fibroblasts in mature SM, but in fibrosis conditions, it may be produced by other cell types, such as myofibers, MSCs, inflammatory cells, or endothelial cells. Fibrosis is the aggregation of excess ECM components, common in most myopathies. Fibrosis in SM occurs during myopathy, aging, and diabetes, usually characterized by increased endomysium as well as perimysium [12]. Expression of collagen I, III, and IV is reported to be increased throughout diet-induced insulin resistance (IR) [35]. Both the murine model and human patients show that the level of SM collagen is higher in insulin resistance (IR) [36]. In a comparative study, total collagen content was observed to increase in obese insulin-resistant individuals compared with lean individuals [37]. Diabetes-induced alterations in SM concern the structure of the BM and the actions of the enzymes responsible for the synthesis of collagen. Gene expression of several collagen types (I, III, IV, V, VI, and XV) was found to be reduced in streptozotocin-induced diabetic mice in a microarray analysis of SM transcriptome. Additionally, mRNA expression of several non-collagenous proteoglycans and glycoproteins was found to be increased in diabetic muscles [38].

### 2.2. Laminin

Laminin is a heterotrimeric glycoprotein and a foremost component of the BM. Laminin-211 (previously named merosin) is the most abundant isoform of laminin in the BM of adult SM. However, other isoforms exist during myogenesis and at junctional regions (e.g., the neuromuscular junction and the myotendinous junction) of the muscle fiber [39]. Laminin-211 is composed of one α2 chain, one β1 chain, and one γ1 chain. The biological functions of the laminins are typically reliant on binding to the cell surface receptors. Two main groups of laminins receptors are known (i.e., integrins and non-integrins).

Integrin α7β1 has been recognized as the main receptor for laminins in adult SM. The α7 subunit is present as α7A and B in adult SM of mouse and human, where it attaches with an integrin β1 splice form (β1D subunit) [40]. Integrin α7Bβ1D is expressed throughout the sarcolemma, while α7Aβ1D expression is limited to junctional sarcolemma [41]. The foremost non-integrin cell surface receptor of laminins in SM is dystroglycan, a central piece of the dystrophin–glycoprotein complex (DGC) [42]. Laminin-211 binds to dystroglycan through O-linked mannose chains of α-dystroglycan (α-DG). α-DG is non-covalently connected to β-dystroglycan, which binds to dystrophin inside the muscle fiber. Other non-integrin receptors of laminin-211 in SM include sulfatides and syndecans [43,44]. Laminin-211 also interacts with several other ECM components, for example perlecan, agrin, and nidogen [39].

### 2.3. ECM Receptors

#### 2.3.1. Integrin

Integrins are the key receptors of most SM ECM components, facilitating mechanical communication between ECM components and cells; they have several critical roles, such as cell attachment, migration, and differentiation [45]. Integrins also facilitate unique bidirectional signaling between ECM and intracellular molecules (“inside-out” and “outside-in” signaling) [46]. Integrins are heterodimeric, having two subunits (α and β). SM express seven α subunits (α1, α3, α4, α5, α6, α7, and αv) in combination with the β1 subunit [47,48].

Collagen and laminin in SM bind mostly with the β1 subunit of integrin [48]. In vertebrates, collagen binds with four integrin receptors that have β1-subunits in association with any of the α1-, α2-, α10-, or α11-subunits [49]. Previously it was observed a reduced activation of focal adhesion kinase (FAK) in insulin-resistant SM of high-fat fed rats that specify the role for integrin-collagen interaction in the expansion of IR [50]. Mice with integrin β1 subunit-deficient striated muscle show IR, as measured by diminishing insulin-mediated glucose uptake and glycogen synthesis in SM and indicated by a reduction in phosphorylation of protein kinase B (AKT) Ser-473 [51]. Downstream signaling of integrins depends on the involvement of the intracellular kinases, FAK and integrin-linked kinase (ILK). Disrupted signaling of integrin and the subsequent inflection of FAK and ILK are found to regulate insulin sensitivity in SM, possibly via altered capillary density [36,52]. As the muscle capillaries are established in direct contact with the ECM, any defect in the recruitment of these capillaries leads to development of SM IR [53]. Zong et al. suggested a connection between abnormal signaling of integrin and the development of T2DM. They observed a decrease of insulin-mediated glucose infusion rate and clearance of glucose in the muscle-specific integrin β1-deficient mice, notwithstanding any changes in the intake of food, weight, glucose (fasting), GLUT4 expression, or insulin levels [36]. Meakin et al. reported that β2-integrins control homeostasis of glucose during high-fat feeding, mostly through actions on SM, to affect the metabolic phenotype in vivo [54]. Furthermore, in an experiment with obese high-fat fed mice, with deletion of α2 integrin from the whole body, a moderate reverse of diet-induced muscle IR was observed, as confirmed by augmented insulin-mediated uptake of glucose through a hyperinsulinemic-euglycemic clamp, and increased insulin signaling [36].

#### 2.3.2. Non-Integrin ECM Receptors

##### CD44

CD44 is a glycoprotein cell surface receptor for a number of ECM components, such as hyaluronan (HA), osteopontin, collagen I, and fibronectin, which are mostly present in the adipose tissue, SM, pancreas, liver, and endothelium. CD44 plays a significant role in the regulation of diverse cellular functions, including cell aggregation, endothelial cell proliferation, and immune cell migration and activation [55]. A genome-wide association study demonstrated that the *cd44* gene is associated with the pathogenesis of T2DM [56]. Hasib et al. hypothesized that high-fat feeding in mice increases HA content and expression of CD44; this activates CD44 signaling and enables muscle capillary rarefaction, successively leading to the development of IR. Interruption of this pathway at several stages, for example, by reduction of HA via PEGPH20 treatment and/or CD44 deletion, helps in reducing IR in SM. Hasib et al. concluded that HA-CD44 signaling might be a probable therapeutic target for IR and T2DM [57].

##### Dystroglycan

Dystroglycan, a transmembrane protein comprising α and β subunits, is a constituent of the dystroglycan complex (DGC) that enables an interaction between the ECM and cytoskeleton of muscle cells. The DGC is a key receptor system for the components of the ECM in SM [58]. Dystroglycan confers stability to myotube by binding to its ligand (laminin-211) during muscle contraction [59]. As dystroglycan is a heavily glycosylated protein, any defect in this glycosylation may lead to several forms of muscular dystrophy [59]. The DGC is involved in essential cell-signaling processes and serves as a binding platform for several ligands, including nitric oxide synthase, which is known to stimulate the transport of glucose. Thus, disruption in the DGC or its components may lead to abnormal insulin signaling in SM fibers and result in altered functionality. Collectively, alterations in the DGC may create changes in glucose metabolism, e.g., IR in the SM of patients with muscular dystrophy [60,61].

##### Syndecans

Syndecans are transmembrane proteoglycans that form a core set of proteins linked to linear carbohydrate chains, known as glycosaminoglycans (GAG) [62,63]. In addition to binding growth factors through the glycosaminoglycan chains, Syndecans can bind directly to ECM molecules [64].

## 3. Skeletal Muscle ECM and Insulin Resistance

SM is the main tissue that regulates glucose homeostasis in the human body; roughly 80% of consumed glucose is taken up by SM and stored in the form of glycogen [65]. A deficiency of glucose consumption in SM is a prominent part of IR, which is known to be an associated risk factor for several disease conditions, of which cardiovascular diseases and diabetes are the most common [66]. Alterations in the composition of the ECM are a common characteristic of IR SM. Increased production of ECM components in the SM is one of the distinguishing features of all prolonged diabetic complications. Typically, increased collagen content is the hallmark of insulin-resistant SM in overweight and diabetic (T2DM) individuals [37]. Likewise, a high-fat diet results in an increase in collagen IV in SM [67].

Glucose homeostasis is sustained by managing glucose production in the liver via glycogenolysis and gluconeogenesis during fasting, supplying glucose to SM through glycogen synthesis and glucose metabolism, a process that occurs to a considerably lesser extent in adipose tissue [68]. The disruption of GLUT4 translocation to the surface membrane in SM and the activation of the transcription factor FOXO1 in the liver impairs the ability of insulin to lower blood glucose levels [68].

**Figure 1:**
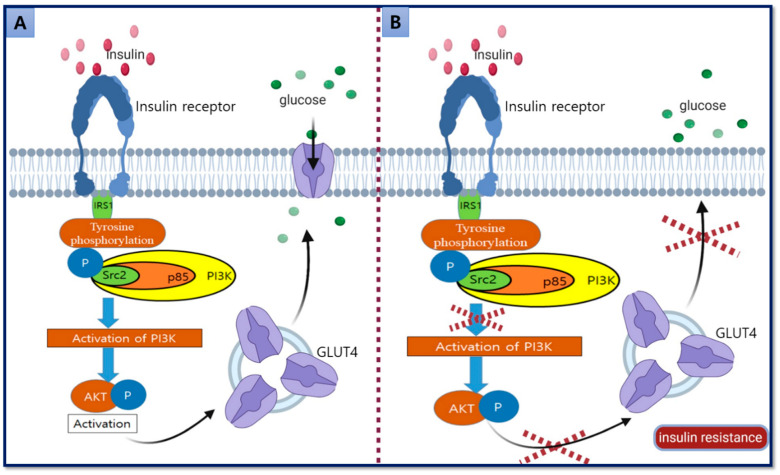
Glucose uptake mechanism via insulin-mediated translocation of GLUT4. (**A**) Insulin binds to the insulin receptor, activating autophosphorylation and PI3K, which activates AKT followed by translocation of vesicles containing GLUT4 from the intracellular compartment to the surface membrane. GLUT4 inserts into the membrane and triggers the uptake of glucose. (**B**) Disturbance in the activation of PI3K and AKT pathways disrupts the GLUT4 translocation that eventually results in the development of insulin resistance (IR) [arrow shows the next step of the process and cross shows the disruption of the process].

Insulin binds to its receptor at the membrane and activates the components of downstream signal transduction pathways, including insulin receptor substrate (IRS)-1, phosphatidylinositide 3-kinases (PI3K), and AKT, which regulate insulin-mediated glucose uptake from the cytoplasm to the plasma membrane via GLUT4. Disturbance in the signal transduction pathways and consequent GLUT4 translocation results in the development of IR and T2DM [69] (Figure 1).

**Figure 2:**
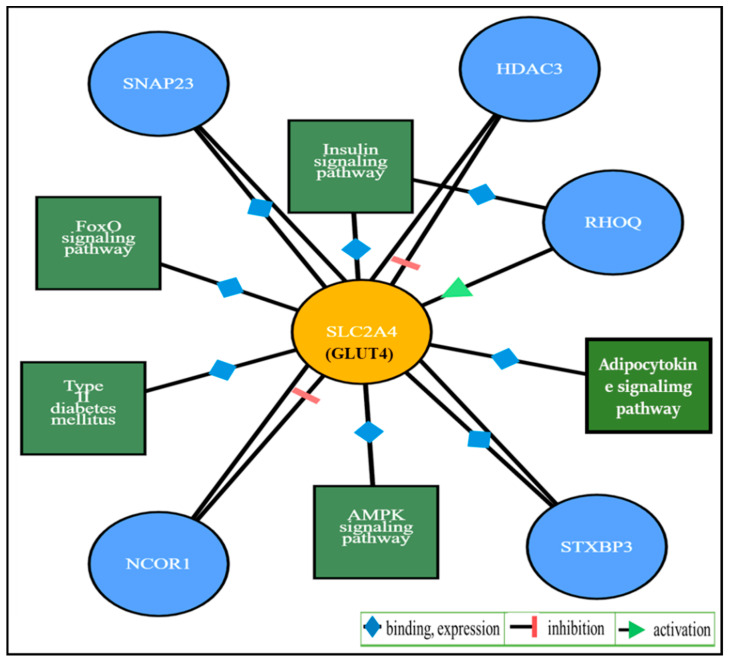
Illustration of interacting pathways and genes with SLC2A4 (GLUT4).

The gene *SLC2A4* (solute carrier family 2, facilitated glucose transporter member 4) encodes GLUT4. An interaction study of the *SLC2A4* gene, predicted by ProteomicsDB [70], shows its relationship with pathways such as T2DM, FoxO signaling, adipocytokine signaling, insulin signaling, and AMPK signaling, as well as interactions with the protein coding genes *SNAP23, HDAC3*, *RHOQ*, *NCOR1*, and *STXBP3*. The interacting pathways for these protein–protein interactions with GLUT4 were assessed by automatic mapping of identifiers using STRING [71] and the functional pathways were determined by KEGG [72] as shown in Figure 2.

In one study, several SM ECM genes (*COL1a1*, *COL4a1*, *COL5a1*, *COL6a1*, *COL6a3*, *SPARC*, and integrin) were found to be upregulated in a group of healthy males whose body weight rapidly increased with compromised insulin sensitivity. Rapid weight gain in these subjects was not due to increased adipose tissue, signifying the role of SM ECM in glucose homeostasis regulation. The investigators concluded that the remodeling of SM ECM is an additional feature of the pathogenesis associated with excess energy and obesity and interrupting this remodeling may result in the progression of metabolic dysfunction [35].

Hyaluronan is a key SM ECM component that creates space between the cells, in addition to several other functions, and increases in high fat diet induced obesity in mice. Elevated hyaluronan levels in SM are associated with SM IR in obesity, probably due to the increased activity of hyaluronan synthase or reduced deprivation by hyaluronidase [73]. A decrease of SM hyaluronan by the injection of pegylated human recombinant hyaluronidase PH-20 (PEGPH20) results in an increase in the dose-dependent glucose infusion rate and SM glucose uptake, as measured by a hyperinsulinemic-euglycemic clamp. These outcomes indicate that ECM hyaluronan may be a possible target for the treatment of IR [73].

## 4. Skeletal Muscle ECM Remodeling and Metabolic Disorders

Skeletal muscle cells dynamically maintain their niche via ECM remodeling, which, together with variations in the expression pattern of the integrin receptors, influences myogenesis [74]. Remodeling of SM ECM is linked to diet-induced IR in several metabolic tissues, especially SM and adipose tissue. Insulin-resistant SM has increased deposition of collagens, both in humans and rodent models [37]. Fibronectin, laminin, and collagen IV synthesis are upregulated by high blood glucose and diabetes, which can lead to thickening of the BM and the development of diabetes-allied microangiopathy [75]. Integrin, a key receptor of ECM molecules, is also involved in the regulation of insulin activity [48]. Collagen I and III were found to be increased in diabetic as well as non-diabetic obese individuals; overfeeding was associated with higher expression of genes linked to IMCT, as well as alterations in intracellular pathways related to ECM receptor [76]. IMCT, cell surface receptors (integrins), and matrix metalloproteinases (MMPs) have been shown to play a role in the progression of IR, particularly in overfeeding conditions [77,78].

ECM remodeling primarily involves the cleavage of the ECM components to regulate the composition, structure, and abundance of ECM. ECM remodeling is also necessary for the secretion of biologically active molecules, for example, growth factors. MMPs are proteolytic enzymes that regulate the homeostasis of myofiber functional integrity by degrading ECM proteins and regulating migration, differentiation, and regeneration of MSCs [79]. MMPs are the key enzymes that degrade the ECM components. Their activity is weak under normal circumstances but elevated during recovery or remodeling in diseased or inflamed tissue [2]. In the SM, only a few MMPs are known to participate in muscle repair and remodeling. Notably, MMP-2 and MMP-9 are prominent in the regenerative process as they are highly expressed after SM damage and aid specific functions to endorse repair and remodeling [80]. MMP-2 and MMP-9 are known as gelatinases, whereas MMP-1, MMP-8, and MMP-13 are known as collagenases. MMP-13 predominantly cleaves collagen I–IV and several other ECM proteins [81]. The expression of MMP-13 is triggered seven days after muscle injury and apparently peaks at around 11 days, signifying its key role in myoblast migration contributing to the secondary effects on differentiation [82]. The roles of different MMPs in SM have been established by earlier studies; MMP-1, MT1-MMP, MMP-2, and MMP-9 actively participate in MSC migration and differentiation in both in vitro and in vivo models [79].

MMPs and tissue inhibitors of metalloproteinases (TIMPs) are known to play a role in the regulation of ECM homeostasis. Degradation of ECM components, mainly collagens, is initiated by MMPs, which can also promote cell migration, proliferation, and differentiation in wounded and diseased SM [79]. Because MMPs are responsible for the depletion of all ECM components, their dysregulation is also associated with the pathology of diabetes and obesity. In high-fat fed mice, MMP-9 activity in SM is decreased and is inversely related to the deposition of muscle collagen but directly to SM IR [36].

**Figure 3:**
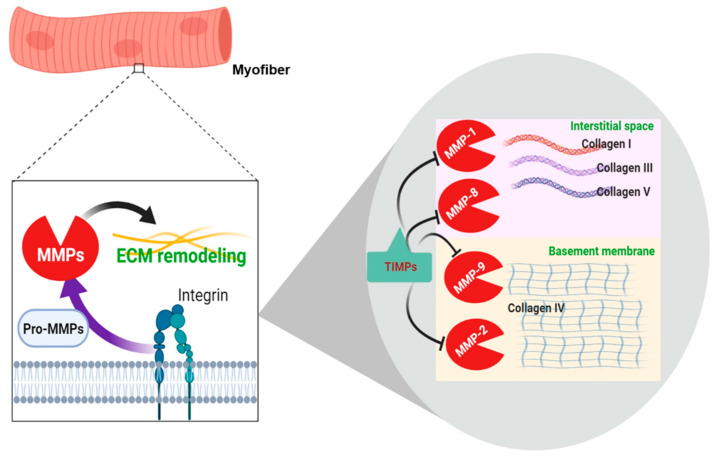
Schematic representation of extracellular matrix (ECM) remodeling in skeletal muscle.

Degradation of collagens through MMPs is fundamental to ECM remodeling [83]. More precisely, MMP-1 and -8 are known to degrade collagen I and III, while MMP-2 and -9 degrade collagen IV [10] (Figure 3).

TIMPs are a family of protease inhibitors (TIMP-1, -2, and -4) that act as inhibitors of all recognized types of MMPs [84]. The N-terminal regions of TIMPs bind to the catalytic domain of MMPs and inhibit their action, while the TIMP C-terminal region may interact with the hemopexin domains of MMP-2 and MMP-9 to stabilize the inhibitor complex. TIMP-1 has been reported to primarily inhibit MMP-1, MMP-3, MMP-7, and MMP-9 [85]. TIMPs, mediated by integrins, can modify cell growth and survival in an MMP-independent manner, e.g., TIMP-2, which controls the expression of beta1 integrin and the size of myotubes produced during myoblast differentiation [86]. TIMPs are frequently activated in response to physical activity along with MMPs, representing the instantaneous stimulation and inhibition of the degradation of ECM components. The activation of MMPs accompanies the activation of TIMPs, which act as “guardians” for the termination of degradation, placing limits on the breakdown of ECM [87].

The expression levels of TIMP-1 and -2 mRNA were found to be increased in the SM of patients with Duchenne muscular dystrophy, a disorder characterized by the inhibition of MMPs and formation of fibrous scar tissue in the diseased muscle [88]. The levels of TIMP-1 and -2 are usually higher in patients suffering from metabolic disorders and T2DM [89]. In an experiment with forty healthy people who were overfed by 1250 kcal/day for 28 days, mRNA levels of collagen I, collagen II, and MMP-2 were considerably increased in SM biopsies without substantial changes in MMP-9, TIMP-1, CD68, or integrin expression. It was concluded from this study that the remodeling of SM ECM happens early in reaction to over-nutrition with a slight (3%) gain in body weight [76].

Disintegrin and metalloproteinase with thrombospondin motifs 9 (ADAMTS-9) is a secreted metalloprotease active against the aggregating proteoglycans versican and aggrecan in ECM. The *ADAMTS9* gene is responsible for the association between the rs4607103 C allele and IR in humans. The SM of individuals carrying the rs4607103 C risk allele has both reduced insulin signaling and augmented levels of ADAMTS-9 expression. Notably, the results of a knockout study and an overexpression study of *Adamts9* in SM in mice indicate that ADAMTS-9 can regulate SM insulin sensitivity [52,90].

In many disease conditions, physical activity has been established to exert beneficial and health-promoting effects. Regular physical activity has been shown to improve insulin sensitivity and thus, enhance metabolic functions in individuals suffering from metabolic disorders like T2DM. In general, exercise increases mechanical stress in the SM, which stimulates and activates the proteases (MMPs/cathepsins) and further enables the remodeling and processing of most ECM components [91,92]. Both MMP-2 and MMP-9 are expressed in different cell types of the human SM, including SM fibers, and both are reported to be upregulated by physical exercise [93]. Exercise was observed to exert a positive effect on TIMP-2 modulation that improved insulin sensitivity [94].

In a screening study for genes involved in ECM organization, development, and degradation, several MMPs (MMP-3, -9, and -15), ADAM metallopeptidases (ADAMTS-1 and -8), collagen (COL7A1 and COL1A1), laminins (LAMB3 and LAMA1), integrins, and other cell adhesion molecules were identified as candidates that were selectively upregulated or downregulated in either old or young individuals. The expression of *COL7A1* decreased in response to resistance training workout, regardless of age, whereas the expression of *ADAMTS1* increased. *MMP* gene expression was also affected by exercise in an age-related manner, as the expression of *MMP9* was augmented in younger subjects but reduced in older subjects, although *MMP15* expression was decreased only in older subjects [95].

## 5. Concluding Remarks and Future Perspectives

Disturbances of ECM homeostasis are associated with several pathological conditions. SM ECM remodeling has been proposed as a feature of the pathogenesis of obesity and metabolic dysfunction. Several ECM components have been shown to be affected by different stages of diabetes. Whether diabetes is linked to muscle weakness is still debated, and whether the changes in ECM-linked pathways are directly involved in this milieu likewise remains to be clarified. The proteolytic and cross-linking cascades of the ECM have critical significance in health and disease and are receiving increased attention from researchers and clinicians. In general, degradation increases disturbance in the arrangement of ECM components in several pathological conditions, including existing diabetic complications. MMPs and TIMPs are crucial in ECM remodeling, and further comprehensive study is needed regarding the signaling mechanism involved in direct or indirect regulation of MMPs in the pathogenesis of SM. Moreover, using other growth factors or non-toxic chemical agents to promote expression of MMPs may prove advantageous for tissue remodeling. It has also been suggested that limiting ECM expansion or targeting the signaling mechanism of integrin receptors may deliver novel opportunities to discover therapeutics for IR and eventually T2DM. Taken together, an effort to improve the characterization of ECM arrangement and metabolism can lead to identification of novel predictive and diagnostic markers and further therapeutic possibilities for metabolic disorders. 

## Abbreviations

ECM	extracellular matrix
SM	Skeletal muscle
GLUT4	glucose transporter type 4
T2DM	type 2 diabetes mellitus
IMCT	intramuscular connective tissue
BM	basement membrane
DPT	dermatopontin
IR	insulin resistance
DGC	dystrophin–glycoprotein complex
FAK	focal adhesion kinase
ILK	integrin-linked kinase
AKT	protein kinase B
MMPs	matrix metalloproteinases
MSCs	muscle satellite cells
TIMPs	tissue inhibitors of metalloproteinases
